# Mediating Role of BMI in the Association Between Obstructive Sleep Apnea and Nonalcoholic Fatty Liver Disease: A Cross-Sectional Mediation Analysis

**DOI:** 10.1155/carj/8606659

**Published:** 2025-11-24

**Authors:** Zhende Luo, Lan Shu, Wei Yu

**Affiliations:** Quality Control Office, Zigong Fourth People's Hospital, Zigong, Sichuan, China

**Keywords:** body mass index, mediation effect, nonalcoholic fatty liver disease, obstructive sleep apnea, structural equation modeling

## Abstract

**Objective:**

To investigate the mediating role of body mass index (BMI) in the association between obstructive sleep apnea (OSA) and nonalcoholic fatty liver disease (NAFLD).

**Methods:**

In this cross-sectional study, 445 adult patients diagnosed with OSA at Zigong Fourth People's Hospital (2022–2024) were initially enrolled. After applying inclusion/exclusion criteria, 309 participants were included. Demographic characteristics and clinical parameters were collected. Correlation analysis and structural equation modeling (SEM) were utilized to assess BMI's mediation effect between OSA and NAFLD.

**Results:**

Correlation analyses revealed significant positive associations of BMI with both OSA (*r* = 0.296, *p* < 0.001) and NAFLD (*r* = 0.225, *p* < 0.001), while OSA and NAFLD were directly correlated (*r* = 0.199, *p* < 0.001). Mediation analysis demonstrated a significant indirect effect of OSA on NAFLD through BMI (*β* = 0.044, 95% CI: 0.004–0.084), accounting for 22.5% of the total effect. These findings remained robust after adjusting for covariates including sex, age, smoking status, hypertension, and diabetes mellitus.

**Conclusion:**

BMI serves as a critical mediator in the OSA–NAFLD relationship, suggesting that integrated weight management strategies should be incorporated into OSA therapy to reduce hepatic risk.

## 1. Introduction

Obstructive sleep apnea (OSA), a prevalent sleep disorder characterized by recurrent upper airway collapse during sleep, leads to intermittent hypoxia (IH) and sleep fragmentation. OSA demonstrates a high global disease burden, with epidemiological studies estimating approximately 1 billion affected individuals worldwide. The condition exhibits an adult prevalence rate of 37% for any OSA severity and 16% for moderate-to-severe OSA. This prevalence escalates substantially in obese populations, where 69% present with varying OSA severity and 32% meet diagnostic criteria for moderate-to-severe OSA [1]. Studies show that for each 1-unit increase in body mass index (BMI), the risk of an elevated apnea–hypopnea index (AHI), a marker of OSA severity, rises by 14% [2]. A 10% weight gain can boost AHI by 32%, and multiply the risk of developing moderate-to-severe OSA by six times. Conversely, a 10% weight loss may lower AHI by 26% [3]. Also, as BMI goes up, the prevalence of OSA shows a parallel upward trend [4].

Nonalcoholic fatty liver disease (NAFLD) is a chronic liver ailment marked by hepatic fat accumulation. It is defined as an increase in liver fat content (steatosis), excluding other known causes like excessive alcohol use, viral hepatitis, or toxins. NAFLD encompasses simple steatosis (without significant inflammation or fibrosis) and nonalcoholic steatohepatitis (NASH, with inflammation and hepatocyte injury). It is one of the most prevalent chronic liver diseases globally, with a prevalence of around 25% [5–7]. Obesity is a major risk factor, present in about 70% of NAFLD patients [8]. However, NAFLD also affects nonobese individuals, with a prevalence of about 40% [6].

A significant epidemiological link exists between OSA and NAFLD. Multiple studies have shown that the prevalence of NAFLD is significantly higher in OSA patients than in the general population, especially among obese individuals, and the severity of OSA is positively correlated with the severity of NAFLD [6, 7, 9]. Research has found that patients with simple snoring and mild, moderate, and severe OSA have rates of hepatic steatosis of 42.86%, 63.5%, 79.4%, and 79.2%, respectively [10]. OSA is also associated with elevated levels of alanine aminotransferase (ALT) [11], with OSA patients having ALT and aspartate aminotransferase (AST) levels increased by approximately 13.3% and 4.4%, respectively. Moreover, NAFLD patients with OSA have a 2.6-fold higher risk of developing liver fibrosis [12].

Obesity, a common risk factor for OSA and NAFLD, impacts their development through multiple mechanisms [8, 10, 13]. First, it causes insulin resistance (IR), a central driver of NAFLD. OSA-related IH and sleep fragmentation worsen IR, boosting hepatic fat deposition and inflammation. Second, obesity and OSA trigger systemic inflammation and oxidative stress, directly damaging hepatocytes and advancing NAFLD. Third, obesity disrupts adipokine balance (e.g., leptin and adiponectin), and OSA exacerbates this imbalance, promoting hepatic steatosis and fibrosis.

Although weight significantly impacts the OSA–NAFLD link, controversies remain. Some studies suggest OSA may influence NAFLD independently of obesity, as their association is seen even in nonobese individuals [14]. Current research on BMI's mediating role between OSA and NAFLD is limited, with analyses not employing bootstrap or structural equation modeling. This study aims to fill this gap, offering evidence for early OSA patient intervention, like weight loss therapy, to prevent NAFLD.

## 2. Materials and Methods

### 2.1. Patients

**Figure 1:**
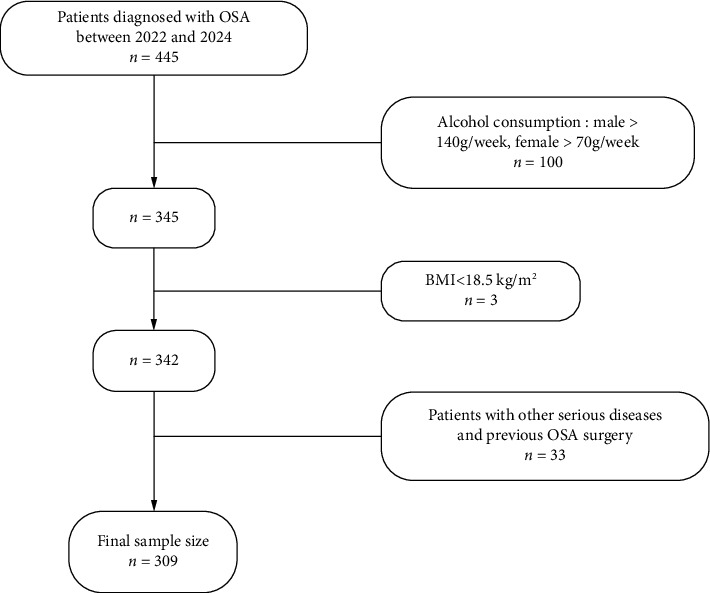
Research object into the flowchart.

This cross-sectional study recruited 445 adults diagnosed with OSA at Zigong Fourth People's Hospital from 2022 to 2024. After applying inclusion and exclusion criteria, the final sample size was 309 (Figure 1). All participants signed informed consent forms and provided basic information and medical history. The study adheres to the Declaration of Helsinki and was approved by the hospital's ethics committee.

Inclusion criteria: (1) aged 18–80, (2) complete basic data, (3) OSA diagnosis via PSG, and (4) available liver imaging report.

Exclusion criteria: (1) other liver diseases besides NAFLD (viral hepatitis, alcoholic liver disease, autoimmune liver disease, etc.); (2) previous weight loss or snoring surgery; (3) long-term continuous positive airway pressure (CPAP) use; (4) malignant tumors, chronic kidney disease, or thyroid dysfunction; and (5) alcohol consumption: > 140 g/week for men, > 70 g/week for women.

### 2.2. Polysomnogram

All patients underwent overnight sleep monitoring for no less than 4 h. According to the standard of the American Academy of Sleep Medicine [15], the population was divided into a mild-to-moderate group (5 times/h ≤ AHI ≤ 30 times/h) and a severe group (AHI > 30 times/h).

### 2.3. General Condition and Imaging Examination

We measured patients' height and weight to calculate BMI and categorized them into three groups in line with the Chinese standard (WS/T 428–2013): normal weight (18.5 kg/m^2^ ≤ BMI < 24.0 kg/m^2^), overweight (24.0 kg/m^2^ ≤ BMI < 28.0 kg/m^2^), and obese (BMI ≥ 28.0 kg/m^2^). We collected patients' medical history, including hypertension, diabetes, and cardiovascular diseases. All laboratory tests, including liver function tests and abdominal ultrasonography, were completed within 48 h of admission to ensure synchronous data assessment. The diagnostic criteria are mainly based on the ultrasound diagnostic criteria recommended in the “Guidelines for the Prevention and Treatment of Metabolic (Non-alcoholic) Fatty Liver Disease (2024)” issued by the Imaging Group of the Chinese Medical Association Hepatology Society. NAFLD was confirmed via imaging and defined as fatty liver in individuals without excessive alcohol consumption.

### 2.4. Statistical Analysis

**Figure 2:**
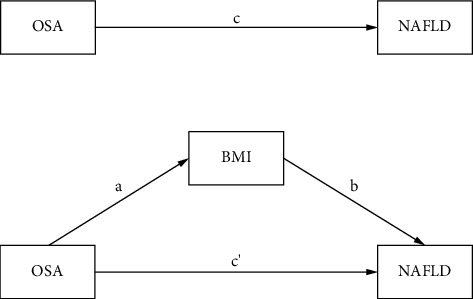
Theoretical model of BMI mediating effect of OSA on NAFLD.

Data were organized in Excel and analyzed using STATA 17.0. We used the chi-square test or ANOVA to analyze differences in indicators across OSA severity groups (mild-to-moderate/severe) and BMI categories (normal/overweight/obese) and performed correlation analysis. We developed a theoretical model (Figure 2) based on relationships between variables. Model fit was assessed with the standardized root mean square residual (SRMR). Bootstrap methods calculated bias-corrected 95% CI for effect sizes; a CI not including 0 indicated significance. The significance level *α* was set at 0.05, with *p* < 0.05 indicating statistical significance.

## 3. Results

### 3.1. The Basic Situation of the Research Object

This study included 309 OSA patients, with severe OSA accounting for 61.5%; 70.6% were male. The average age was 47.52 ± 12.97 years, and 53.4% were aged 40–60. Only 45 patients (14.6%) had a normal weight, while 264 (85.4%) were overweight or obese. 31.4% were smokers, 40.8% had hypertension, 21.0% had diabetes, and 45.3% had NAFLD (Table 1).

### 3.2. Comparison of Patients With Different Degrees of OSA

Among the 309 OSA patients, the mild-moderate OSA group had an average age of 48.18 ± 13.34 years, with 76 male patients, 34 smokers, 41 with hypertension, 18 with diabetes, and 39 with NAFLD. The severe OSA group had an average age of 47.11 ± 12.74 years, with 142 male patients, 63 smokers, 85 with hypertension, 47 with diabetes, and 101 with NAFLD. Comparison of the three groups revealed differences in gender, BMI, diabetes, and NAFLD (*p* < 0.05) but no significant differences in age, smoking status, and hypertension (*p* > 0.05) (Table 2).

### 3.3. Comparison of Patients With Different Degrees of Weight

Of the 309 OSA patients, the normal BMI group had 62.2% males, an average age of 52.93 ± 13.48 years, 26.7% smokers, 37.8% with hypertension, 13.3% with diabetes, 17.8% with NAFLD, and 35.6% with severe OSA. The overweight group had 72.7% males, average age of 48.25 ± 12.23 years, 27.3% smokers, 35.5% with hypertension, 22.7% with diabetes, 44.5% with NAFLD, and 57.3% with severe OSA. The obese group had 71.4% males, average age of 45.43 ± 12.89 years, 35.7% smokers, 45.5% with hypertension, 22.1% with diabetes, 53.9% with NAFLD, and 72.1% with severe OSA. Comparison among the three groups found differences in age, NAFLD, and OSA (*p* < 0.05) but no significant differences in gender, smoking, hypertension, or diabetes (*p* > 0.05) (Table 3).

### 3.4. Analysis of Relationship

As given in Table 4, OSA was positively correlated with BMI, diabetes, and NAFLD (*r* = 0.2963, 0.1148, 0.1993, *p* < 0.05) and negatively correlated with gender (*r* = −0.1161, *p* < 0.05). BMI was positively correlated with OSA and NAFLD (*r* = 0.2963, 0.2247, *p* < 0.001) and negatively correlated with age (*r* = −0.1793, *p* < 0.01). NAFLD was positively correlated with OSA and BMI (*r* = 0.1993, 0.2247, *p* < 0.01).

### 3.5. The Mediating Role of BMI in OSA on NAFLD

**Figure 3:**
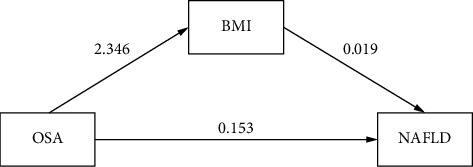
Pathway map of BMI mediating effect of OSA on NAFLD.

A structural equation model was built based on the theoretical hypothesis. Since robust standard errors were used, traditional fit indices were not reported. Model quality was assessed using SRMR and bootstrap confidence intervals. Results showed that SRMR was less than 0.08 and the confidence intervals did not include 0, indicating a reliable model (Figure 3). After bootstrap analysis (500 replications), BMI was found to have a mediating effect between OSA and NAFLD: (1) Indirect effect: Severe OSA indirectly increased NAFLD risk by elevating BMI (*β* = 0.044, 95% CI = 0.004–0.084, *p*=0.030), accounting for 22.5% of the total effect. (2) Direct effect: After controlling for BMI, severe OSA still directly increased NAFLD risk (*β* = 0.153, 95% CI = 0.039–0.266, *p*=0.008), accounting for 77.5% of the total effect. (3) Total effect: The total effect of OSA on NAFLD was significant (*β* = 0.197, 95% CI = 0.088–0.306, *p* < 0.001). The results indicate that BMI is a partial mediator between OSA and NAFLD (Table 5).

## 4. Discussion

This study, for the first time, systematically verifies the mediating effect of BMI between OSA and NAFLD using the bootstrap method. The results show that the mediating effect of BMI accounts for as high as 22.5% (*β* = 0.044), indicating that 22.5% of the impact of OSA on NAFLD is mediated by BMI. This finding offers a new explanatory framework for the pathophysiological link between OSA and NAFLD.

We found a positive correlation between OSA and BMI (*r* = 0.2963, *p* < 0.001). In the mediation analysis, the path coefficient (a path) of OSA to BMI was 2.346 (*p* < 0.001), indicating a positive impact of OSA severity on BMI. Specifically, a one-unit increase in OSA severity is associated with a 2.346-unit increase in BMI. The mechanisms may be multifaceted: (1) Energy metabolism imbalance and fat accumulation: Chronic intermittent hypoxia (CIH) in OSA patients activates the hypoxia-inducible factor (HIF) pathway, inhibits mitochondrial *β*-oxidation, reduces fat decomposition efficiency, and promotes fat synthesis [16]. Additionally, continuous sympathetic nerve activation in OSA patients lowers the resting metabolic rate (RMR), exacerbating positive energy balance and visceral fat accumulation [17]. CPAP treatment, while improving hypoxia, has variable effects on energy metabolism regulation. Some patients show no significant RMR improvement posttreatment, suggesting potential irreversibility of energy metabolism disorders [18]. (2) Hormonal dysregulation and hyperphagia: OSA disrupts hormonal balance through multiple pathways. IH reduces leptin sensitivity and increases ghrelin secretion, leading to hyperphagia and increased caloric intake [16, 19]. Sleep fragmentation decreases slow-wave sleep, suppressing growth hormone (GH) secretion peaks, which weakens fat decomposition and muscle anabolism [17]. (3) Reduced physical activity and behavioral compensation: Daytime sleepiness and fatigue in OSA patients significantly lower physical activity levels, reducing energy expenditure [20, 21]. Long-term sleep deprivation may also trigger emotional eating or night-eating behaviors, further increasing caloric intake [16]. Moreover, genomewide association studies (GWAS) have identified shared genetic loci between OSA and BMI, such as the *FTO* gene. These loci may promote a vicious cycle by regulating appetite, fat distribution, and hypoxia sensitivity, making individuals with obesity-prone genes more susceptible to fat accumulation in OSA-induced hypoxic conditions [22].

In our study, BMI was linked to NAFLD (*r* = 0.2247, *p* < 0.001). The path coefficient (b path) of BMI to NAFLD was 0.019 (*p*=0.012), indicating a positive relationship where each 1-unit BMI increase is associated with a 0.019-unit increase in NAFLD on average. BMI elevation promotes NAFLD development via multiple pathways, including dyslipidemia, IR, epigenetic regulation, chronic inflammation, and energy imbalance. Obesity-induced adipose dysfunction, shown as adipocyte hypertrophy and hyperplasia, triggers chronic low-grade inflammation. This inflammation, marked by imbalanced TNF-α, IL-6, adiponectin, and leptin, promotes hepatic steatosis and IR, a core NAFLD pathogenesis [23]. IR reduces hepatic glucose uptake and utilization while boosting lipolysis, increasing free fatty acid (FFA) influx to the liver, and worsening steatosis. Kuang et al. [24] found that patients with BMI ≥ 30 kg/m^2^ have 2.3 times higher liver fat content than those with normal BMI. Elevated BMI also drives adipose tissue macrophages to proinflammatory M1 polarization, releasing TNF-α and IL-6, which activate hepatic Kupffer cell TLR4 pathways, exacerbating hepatocyte oxidative damage. Multidisciplinary lifestyle interventions (diet + exercise) can lower serum IL-6 by 34% and reduce liver fat by 28% in obese patients (BMI ≥ 40 kg/m^2^) [25]. Hagström et al. [26] found in a cohort study that higher maternal BMI increases offspring NAFLD risk via epigenetic mechanisms (e.g., DNA methylation). Each 5 kg/m^2^ increase in maternal BMI raises offspring NAFLD risk 1.5-fold (OR = 1.52, 95% CI: 1.21–1.91).

Our study confirms that BMI is a key mediator in the OSA–NAFLD link, yet OSA has a direct effect independent of BMI, with a path coefficient *c* of 0.153 (*p*=0.010). This implies other mechanisms like oxidative stress and gut microbiota dysbiosis are at play. The direct effect of OSA on NAFLD, independent of BMI, is widely accepted. In OSA patients, recurrent airway collapse causes IH, which activates hepatic HIF-1*α*, upregulating lipid synthesis genes (e.g., SREBP-1c), and downregulating fatty acid oxidation (e.g., PPARα), leading to hepatic lipid accumulation. This effect is significant even in BMI-matched OSA patients, indicating IH directly impairs hepatic metabolic function [8, 9, 27]. Animal models show nonobese mice exposed to IH have increased hepatic lipid deposition, correlated with hypoxia severity [8]. OSA induces systemic oxidative stress and proinflammatory cytokine release (e.g., IL-6, TNF-α) via IH, activating hepatic Kupffer cells and promoting intrahepatic inflammation and fibrosis. In Asian population studies, upregulated expression of inflammatory genes (e.g., TLR4, NF-κB) in OSA patients is associated with NAFLD severity, independent of BMI [28]. Clinically, OSA patients, even without obesity, show a significant increase in histological activity index (HAI) in liver biopsies [29]. The recurrent nighttime awakenings and hypoxia caused by OSA activate the sympathetic nervous system, increasing adrenaline release. This directly stimulates hepatic stellate cells (HSCs) to produce collagen, promoting hepatic fibrosis [6]. Sleep fragmentation causes circadian rhythm disruption, interfering with the expression of core circadian genes (e.g., BMAL1), which affects the periodic regulation of lipid metabolism genes in the liver, leading to an imbalance between lipid synthesis and breakdown. This effect is also observed in nonobese models [6, 27]. Patients with OSA often have leptin resistance and decreased adiponectin levels. Together, these factors promote hepatic lipid deposition and IR. In BMI-matched cohorts, serum adiponectin levels in OSA patients are negatively correlated with hepatic fat content, and CPAP treatment can improve adiponectin levels [9, 29]. Therefore, OSA can directly promote the progression of NAFLD, independent of BMI, through multiple pathways including IH, inflammation, sympathetic overactivity, adipokine imbalance, and circadian disruption.

In this study, the bias-corrected bootstrap method (with 500 resamples) was used to effectively control Type I errors. Compared with the traditional causal stepwise method (Baron and Kenny method), it shows better robustness for nonnormally distributed data. For the first time, the contribution of BMI was quantified through a mediation model. Also, by incorporating covariates, the internal validity of the effect estimates was significantly enhanced. Nevertheless, there are certain limitations to this study. The cross-sectional design cannot entirely rule out reverse causality (for instance, NAFLD might worsen OSA). The temporality needs to be confirmed via Mendelian randomization or intervention trials (such as changes in BMI/liver fat before and after CPAP treatment). The absence of potential mechanistic variables like IR and inflammatory markers might lead to an underestimation of the mediated effect. The sample was primarily from hospital-based populations, posing Berkson's bias risk, and the results' generalizability needs verification in community cohorts. Furthermore, this study could not collect and adjust for potential confounders such as physical activity levels, dietary patterns, and socioeconomic status, all of which may influence both BMI and the development of NAFLD. Future prospective cohort studies that systematically collect these lifestyle data are needed to more precisely estimate the mediating role of BMI in the OSA–NAFLD relationship. Moreover, the relatively small sample size should be addressed by including more participants in future work.

In summary, our study, using a robust statistical model, confirms that BMI is a significant mediator between OSA and NAFLD. This finding not only deepens our understanding of the metabolic complications of OSA but also provides a theoretical basis for developing multitarget intervention strategies based on weight management. In clinical practice, intensive weight management may prevent NAFLD in over 22.5% of obese OSA patients, so weight loss should be a key goal in treating severe OSA. Also, OSA patients with BMI > 24.0 kg/m^2^ and liver dysfunction should be prioritized for ultrasonography or liver elastography to improve early NAFLD diagnosis.

## 5. Conclusions

This study confirms that BMI is a core mediator in the association between OSA and NAFLD, supporting early interventions like weight loss therapy for NAFLD prevention in OSA patients. Clinically, integrating weight management with OSA treatment is recommended to reduce liver disease risk.

## Figures and Tables

**Table 1:** General description of 309 patients with OSA.

Variable	Classification	*n*	%
Gender	Male	218	70.6
	Female	91	29.4
Age (years)	< 40	91	29.4
	40–60	165	53.4
	≥ 60	53	17.2
Smoking	Yes	97	31.4
	No	212	68.6
BMI (kg/m^2^)	[18.5–24.0)	45	14.6
	[24.0–28.0)	110	35.6
	≥ 28.0	154	49.8
Hypertension	Yes	126	40.8
	No	183	59.2
Diabetes	Yes	65	21.0
	No	244	79.0
NAFLD	Yes	140	45.3
	No	169	54.7
OSA	Mild-moderate	119	38.5
	Severe	190	61.5

**Table 2:** Comparison of patients with different degrees of OSA.

Variable	All (*n* = 309)	Mild-to-moderate group (*n* = 119)	Severe group (*n* = 190)	*P*
Gender				0.041
Male	218	76	142	
Female	91	43	48	
Age (years)	47.52 ± 12.97	48.18 ± 13.34	47.11 ± 12.74	0.479
BMI (kg/m^2^)	28.27 ± 4.19	26.70 ± 3.45	29.25 ± 4.32	< 0.001
Smoking				0.398
Yes	97	34	63	
No	212	85	127	
Hypertension				0.073
Yes	126	41	85	
No	183	78	105	
Diabetes				0.044
Yes	65	18	47	
No	244	101	143	
NAFLD				< 0.001
Yes	140	39	101	
No	169	80	89	

**Table 3:** Comparison of patients with different degrees of weight.

Variable	All (*n* = 309)	Normal group (*n* = 45)	Overweight group (*n* = 110)	Obesity group (*n* = 154)	*P*
Gender					0.404
Male	218	28	80	110	
Female	91	17	30	44	
Age (years)	47.52 ± 12.97	52.93 ± 13.48	48.25 ± 12.23	45.43 ± 12.89	0.002
Smoking					0.263
Yes	97	12	30	55	
No	212	33	80	99	
Hypertension					0.240
Yes	126	17	39	70	
No	183	28	71	84	
Diabetes					0.387
Yes	65	6	25	34	
No	244	39	85	120	
NAFLD					< 0.001
Yes	140	8	49	83	
No	169	37	61	71	
OSA					< 0.001
Mild-to-moderate	119	29	47	43	
Severe	190	16	63	111	

**Table 4:** Correlation matrix.

Variable	BMI	NAFLD	OSA	Gender	Age	Hypertension	Diabetes	Smoking
BMI	1.0000							
NAFLD	0.2247^∗∗∗^	1.0000						
OSA	0.2963^∗∗∗^	0.1993^∗∗∗^	1.0000					
Gender	−0.0129	0.0252	−0.1161^∗^	1.0000				
Age	−0.1793^∗∗^	−0.0705	−0.0404	0.2876^∗∗∗^	1.0000			
Hypertension	0.1023	0.0121	0.1018	0.0707	0.2635^∗∗∗^	1.0000		
Diabetes	0.1058	0.1045	0.1148^∗^	0.0149	0.2423^∗∗∗^	0.2342^∗∗∗^	1.0000	
Smoking	0.0580	0.0287	0.0481	−0.4370^∗∗∗^	−0.0269	0.0205	0.0444	1.0000

^∗∗∗^ *p* < 0.001.

^∗∗^ *p* < 0.01.

^∗^ *p* < 0.05.

**Table 5:** BMI mediates the effect of OSA on NAFLD.

Effect type	*β*	*Bootstrap std. err.*	*P*	95% CI	%
Total effect	0.194	0.056	< 0.001	[0.088,0.306]	100
Direct effect	0.153	0.058	0.008	[0.039,0.266]	77.5
Indirect effect	0.044	0.020	0.030	[0.004,0.084]	22.5

## Data Availability

Data will be made available upon request to the corresponding author.
